# Improving the distribution of antiretroviral medicines through centralised dispensing: perspectives of HIV+ patients and nurses at a chronic dispensing unit in Ekurhuleni, Gauteng Province, South Africa

**DOI:** 10.11604/pamj.2023.45.156.35405

**Published:** 2023-08-11

**Authors:** Virginia Apandju Osako Ngende, Burt Davis

**Affiliations:** 1 Department of Health, Ekurhuleni Chronic Dispensing Unit, Gauteng province, Johannesburg, South Africa,; 2 Africa Centre for HIV/AIDS Management, Faculty of Economic and Management Sciences, Stellenbosch University, Stellenbosch, South Africa

**Keywords:** Central dispensing unit, antiretroviral medicines, HIV, effectiveness, service delivery, patient satisfaction, nurse satisfaction, quality improvement, Ekurhuleni

## Abstract

**Introduction:**

South Africa’s public healthcare facilities are continuously inundated with arduous challenges. A Chronic Dispensing Unit (CDU) can help to alleviate these challenges by reducing the workload of nurses at Primary Health Care clinics, decrease patient waiting time, and enhance accessibility of antiretroviral treatment (ART) for HIV+ patients through the dispensing, packaging, and distribution of chronic medicines to stable patients. Determining the effectiveness of a CDU is therefore critical as it can benefit both the patients and the CDU as a service provider. This study ascertained the efficiency of the Germiston CDU in Ekurhuleni, Gauteng Province, in distributing ART to clinics in order to make possible recommendations for quality improvement. No such study has so far been conducted at this CDU.

**Methods:**

an exploratory quantitative research design was adopted. Data collection techniques consisted of researcher-assisted and self-administered questionnaires. The sample included 60 patients and 11 nurses who volunteered to participate in the study.

**Results:**

main findings showed that patient participants believed there was a noteworthy reduction in patients’ waiting time at clinics. They were also highly satisfied with the CDU’s level of service delivery. Responses from nurse participants indicated an increase in accessibility of ART since the induction of the CDU. However, emerged challenges linked to CDU service delivery warrant a closer inspection of the CDU processes as it revealed shortfalls within the system that may hamper quality of service delivery.

**Conclusion:**

in general, services rendered by CDU were deemed effective. However, as a recommendation, challenges linked to CDU service delivery must be addressed.

## Introduction

Despite its laudable progress towards the fight against HIV and AIDS since the rollout of antiretroviral treatment (ART) in 2004, South Africa´s public healthcare facilities are continuously inundated with arduous challenges. The following are enumerated by Haseeb [1] as points of contention: inadequate healthcare workers, high numbers of patients, long waiting queues and the management of various chronic diseases. Reda [2] and Hwang [3] further claim that limited availability and accessibility of ART disrupt patients’ adherence to treatment. When coupled with an estimated figure of 7.7 million individuals living with HIV in South Africa [4], the overall burden experienced at public healthcare facilities is exponentially exacerbated. This adversely impacts on the optimal function of the supply chain. Consequently, one concept aimed to remedy accessibility of ART, improve patient waiting time and decongest healthcare facilities, is the establishment of a central dispensing unit (CDU) (also referred to as a chronic dispensing unit).

The fundamental role of a CDU is to collect prescriptions of stable patients on long-term therapy from clinics within a district, dispense and package their medicines, and ultimately deliver ready-packed medicines to their respective clinics. According to Magadzire *et al*. [5], a CDU is an essential and innovative intervention for enhancing access to chronic medicines. Similarly, Du Toit [6] remarked that centralised dispensing programs such as a CDU have successfully decongested healthcare facilities and improved access to chronic medicines. The premise of this paper was to investigate the effectiveness of the Germiston CDU in the distribution of ART. This was conducted from the perspective of CDU-enrolled patients and Primary Health Care (PHC) nurses at four PHC clinics in Ekurhuleni health district in the Gauteng province of South Africa.

**The Germiston CDU:** the City of Ekurhuleni Metropolitan Municipality is in the east region of Gauteng province, South Africa, and has a population of 3.97 million people [7]. As per the 2018/2021 District Health Plan, the Health District of Ekurhuleni is committed to providing and improving the quality of healthcare for its residents [8]. Enhancing the quality of healthcare services rendered to patients was the impetus for initiatives such as the Germiston CDU [9], which specializes in the dispensing of chronic medicines in the health district of Ekurhuleni. Four PHC clinics were first registered at the Germiston CDU when it was launched in 2015. Specifically, the Katlehong North Clinic (KNC), Mary Moodley Memorial Clinic (MMMC), Daveyton Main Clinic (DMC), and Phola Park Clinic (PPC). Shortly after its inauguration, the CDU underwent an expansion to accommodate the increasing demand for enrolment of additional clinics within the district. The CDU establishment in Ekurhuleni currently renders pharmaceutical services to a total of 93 clinics.

**Figure 1:**
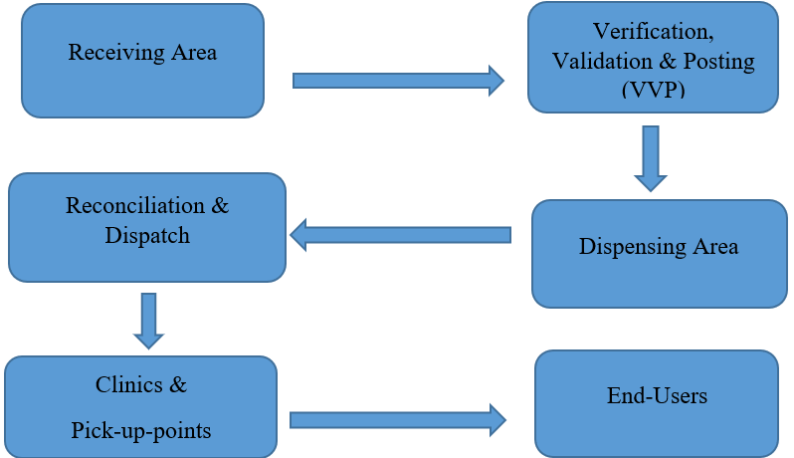
a graphic overview of the operational processes of Germiston CDU from the receiving area to the end-user

**How Germiston CDU works:** the main function of Germiston CDU is to collect prescriptions of stable patients on long-term therapy from clinics within the district; dispense and package their medicines; and ultimately deliver ready-packed medicines to their respective clinics. From the clinic, CDU parcels are delivered to pick-up points (i.e., a designated place approved by the National Department of Health for the collection of patients´ chronic medicines [10]. As Figure 1 shows, the Germiston CDU comprises various subunits with different functions that are sequentially interdependent: i) the “receiving area” is the entry point of the workflow at Germiston CDU. The main objective of this department is to ensure proper receiving, registration, and filing of prescriptions received from facilities; ii) the “Verification, Validation, and Posting (VVP)” department ensures proper verification, capturing, posting, and printing of patients´ medicine labels as per pharmacy regulations. A four-week turnaround time is applied for the processing of prescriptions, providing sufficient time until delivery; iii) the “dispensing area” ensures the application of proper dispensing techniques when handling patients´ prescriptions. The dispensing process entails picking of medication according to the prescription; labeling of medication; checking and signing of prescriptions as well as ensuring statistical record keeping; iv) the “reconciliation and dispatch” department ensures proper reconciliation between prescriptions received and parcels produced. Any discrepancy encountered is immediately rectified before sealing boxes. Boxes of patients´ ready-packed medicines are dispatched according to the clinics´ weekly delivery schedule; v) parcels of medicines are received by nurses at clinics and pick-up points and then collected by end-users (patients).

**The importance of investigating the effectiveness of the Germiston CDU:** the acquisition of data about a CDU´s service delivery from the perspective of patients and nurses serves as a qualitative indicator of the program´s performance [11]. More specifically, the waiting time experienced by patients at healthcare facilities serves as one of the determining factors for quality service delivery. According to Oche and Adamu [12], if health consumers spend a significant amount of time at clinics waiting for services from healthcare professionals, it adversely impacts patients´ satisfaction with the services received. Therefore, determining the effectiveness of a CDU is critical as it can benefit both the patients and the CDU as a service provider. Furthermore, the detection of possible defects in the CDU processes can foster the development of a refined service delivery system, which in turn, could increase health consumers´ satisfaction. For these reasons, a study to ascertain the efficiency of the Germiston CDU is of utmost importance, especially in light of the fact that since its inauguration in 2015, no such study has so far been conducted at this CDU.

To this effect, the main aim of this study was to investigate the effectiveness of the Germiston CDU in distributing ART to clinics. Specific objectives were to: (1) establish the satisfaction level of patients and nurses with regard to service delivery at the CDU; (2) record any changes in the waiting times of patients before and after CDU implementation, and (3) identify any challenges experienced by patients and nurses related to the CDU program, in order to determine potential shortcomings and make feasible recommendations toward the improvement of systems and processes of the Germiston CDU.

## Methods

An exploratory quantitative research design was adopted.

**Study site:** the study was conducted at the four mentioned pioneered PHC clinics i.e. KNC, MMMC, DMC, and PPC. The computerized database management system utilized at these PHC clinics is known as TIER.Net v1.12.6.0. At the time of the study, the database reflected the number of CDU-enrolled patients per study clinic as follows: 1956 patients from KNC, 2159 patients from MMMC, 2158 patients from DMC, and 2432 patients from PPC. In total, there were 8705 patients enrolled at the four clinics. The CDU-enrolled patients obtain their chronic medicines from their respective clinics or selected pick-up points.

**Participants:** two target groups from the four clinics namely; CDU-enrolled patients and nurses who are facilitating the CDU program were targeted for inclusion in the study.

***CDU-enrolled patients:*** a total of 15 participants from each of the four clinics were selected, adding up to a total sample size of 60 participants. The study was small in scale and the aim was not to make inferences about the population, hence the sample was deemed as sufficient. The inclusion criteria for patient participants entailed that they had to be: 1) diagnosed with HIV; 2) on ART for more than six months and stable; 3) enrolled in the CDU program and receiving chronic medicine as ready-patients parcels; and 4) registered at one of the four clinics (KNC, PPC, MMMC or DMC).

The Stat Trek Random Number Generator was administered to draw a random sample [13]. The random selection process was repeated until the desired number of participants was secured. Patients who were present on the day of the study at the various clinics were targeted for random inclusion. All participants who were randomly selected participated voluntarily and gave their informed consent in writing. In terms of demographics, all participants were Black Africans, of which 67% (n=40) of the participants were female and 33% (n=20) of the participants were male. The age distribution among participants ranged from 21 to 65 years old, with a mean age of 43 years old. In terms of the educational background of participants: 21% (n=13) completed primary school; 76% (n= 46) completed secondary school (matric) and 2% (n=1) completed tertiary school.

***Nurses:*** a total of 11 nurses from each of the four clinics were selected to participate in the study. The inclusion criterion for nurse participants was that they had to be involved in the enrolment of patients in the CDU program at the clinics of study interest. A convenience sample of nurses who were present on the day of the study at the four clinics was applied. All participants participated voluntarily and gave their informed consent in writing. With regards to demographics, all nurse participants (n=11) were Black Africans and 100% female. The age distribution among participants ranged from 27 to 47 years old, with a mean age of 37 years old. In terms of the educational background of participants: 22% (n=2) were enrolled nurses and 78% (n=7) were professional nurses.

**Materials:** data collection techniques consisted of researcher-assisted and self-administered questionnaires. Two researcher-designed questionnaires with both open-ended and closed-ended questions were developed for each target group. All data collection instruments were presented in English and IsiZulu. The English version of the questionnaire was translated in IsiZulu by the research assistant and both versions (English and IsiZulu) were given to an independent bi-lingual expert (qualified language translator) to appreciate the accuracy of the translation.

**Measures:** questionnaire variables included the level of service delivery and changes in patient waiting time. We also wanted to identify potential challenges experienced by patients and nurses related to the CDU program. Items to measure the level of service delivery were researcher-developed in accordance with the study objectives. The items purposely contained key elements that would determine whether the Germiston CDU is efficient in rendering services to clinics within the district. Items included, among others, “decrease in patient waiting time”, “prompt delivery of CDU parcels” and “satisfaction with CDU service delivery”. All items used a dichotomous scale (yes/no) and offered participants the opportunity to elaborate on their responses if “no” was selected. The differences between the items to measure the level of service delivery for patients and for nurses are attributed to the different roles each party plays within the program.

To measure changes in patient waiting time, participants were asked to estimate how long they had to wait to receive medication before and after the implementation of the CDU. The question on patient waiting time in the questionnaire consisted of three optional responses (i.e. 0-3 hours, 3-5 hours, and 5-8 hours) of time spent at the clinic. Patient waiting time was computed from the time of the patient´s arrival at the clinic to the time of the patient´s departure from the clinic. According to the South African National Department of Health [14], patient waiting time is defined as “*the time that the patient spends waiting for service(s) in a facility per visit and is calculated from the time the patient enters the facility (taking into consideration the official opening time of a facility) to the time the patient leaves the facility*.”

As mentioned, participants were offered the opportunity to elaborate on their responses if “no” was selected. The aim was to generate a holistic view of the reasoning behind the “no” responses in order to identify potential challenges experienced by patients and nurses. The content validity of each instrument was appreciated by external research experts. According to Christensen *et al*. [15], the judgment of multiple experts has more value than that of one expert, when appreciating the content validity of instruments. The process involved examining each item and verifying its relevance to the research aim and objectives.

**Study procedure:** data were collected between September 2019 and November 2019 from the clinics of study interest. At the different clinics, patient participants were approached by the principal researcher in the waiting room. The nature and purpose of the study were explained to patient participants. A random selection process was administered until the desired number of participants was secured. Willing participants were given consent forms to complete and sign. They completed the questionnaires in a private room at the clinic with the help of the principal investigator, where necessary. In cases of a language barrier, a research assistant assisted in IsiZulu using the isiZulu version of the questionnaire. Self-administered questionnaires were given to nurse participants present and available during the timeframe of data collection, to complete in their own time. The questionnaires were retrieved the following day.

**Statistical analysis:** data was captured using Microsoft Office 365 (Excel) with SPSS version 26. Statistical analyses included descriptive statistics. The themes emerging from the open-ended sections of the questionnaires were identified and documented using a thematic content analysis approach. A numerical value was allocated to each emerging theme.

**Ethical considerations:** prior to commencing the research, approvals from the National Health Research Database (NHRD), Ekurhuleni Health District Research Committee (EHDRC), and Health Research Ethics Committee (HREC) of Stellenbosch University (HREC ref #: S19/06/110), were obtained. The nature of the research and its potential benefits were explained to all relevant stakeholders. Willing participants were given information leaflets about the research and consent forms before completing the self-administered questionnaire. Volunteered participants were also informed that they could withdraw from the study should they feel the need to, at any time, without consequences. It was clearly stipulated that data collected from the study would be dealt with considerable care, anonymity, and confidentiality.

## Results

Table 1 shows that the level of CDU service delivery was perceived as effective across the four clinics, with the majority of participants (87% +) answering affirmatively in regard to the different items measuring this variable. The only exception was at the DMC clinic in relation to the item on incidences where parcels were not delivered at the clinic. Here, 40% of participants (n= 6) indicated that they had encountered this problem. Table 2 shows a marked decrease in the waiting times at all four clinics since the CDU implementation. Prior to the implementation of the CDU, all participants indicated that they waited for at least 3 hours to receive their medication, with an average waiting time of ±5h30m. However, after the implementation of the CDU, the majority of the participants (n=5) indicated that their waiting time now varied between not having to wait at all to have to wait no more than 3 hours, with an average waiting time of ±1h45m. Table 3 shows challenges related to service delivery experienced by patients. Although the CDU service delivery was deemed effective, some of the highlighted challenges were “short collection time-frame for CDU parcels” as declared by 35% (n = 21) of participants; “late delivery of CDU parcels at pick-up-points” as stated by 25% (n = 15) of participants and “prolonged waiting period at pick-up-points” as confirmed by 16.7% (n =10) of respondents. Long waiting time at pick-up points was predominantly experienced at MMMC, as 9 out of the 10 responses were recorded at this clinic alone.

**Table 1:** patients´ responses related to CDU service delivery per clinic (N=60)

Items	PPC (n=15)		KNC (n=15)		MMMC (n=15)		DMC (n=15)	
	YES (%)	NO (%)	YES (%)	NO (%)	YES (%)	NO (%)	YES (%)	NO (%)
I receive my CDU parcels every two months at the clinic.	100	0	100	0	87	13	93	7
I receive my CDU parcels on the day I am supposed to collect them.	93	7	87	13	87	13	87	13
I have encountered incidences of parcels not being delivered	20	80	87	13	27	73	40	60
I have encountered incidences of drugs “out of stock”	0	100	0	100	7	93	0	100
I have received the wrong medication	0	100	13	87	13	87	0	100
There has been a decrease in patients´ waiting time at the clinic since the implementation of CDU	93	7	100	0	80	20	87	13
I am satisfied with the delivery of my CDU parcels	87	13	100	0	80	20	87	13

CDU: chronic dispensing unit; PPC: Phola Park Clinic; KNC: Katlehong North Clinic; MMMC: Mary Moodley Memorial Clinic; DMC: Daveyton Main Clinic

**Table 2:** patient waiting time at the four clinics before and after CDU implementation (N=60)

Hours (range of hours)	Number (#) of patients (N=60)	
	Before CDU	After CDU
0 – 3 hours	0	55
3 – 5 hours	42	4
5 – 8 hours	18	1

CDU: chronic dispensing unit

**Table 3:** challenges experienced by patients at the four clinics with regard to the chronic dispensing unit (CDU) program (N=60)

Challenges	Frequency (n)	Percentage (%)
The collection timeframe for CDU parcels at pick-up-points is short and limited to weekdays only	21	35
Late delivery of CDU parcels at the pick-up-points	15	25
On the collection date, CDU patients with acute conditions must queue to be seen by the clinician, at the clinic	13	21.7
Long waiting times at pick-up-points	10	16.7
Patients do not receive medication as ready-packed parcels	9	15
CDU parcels not delivered on time at clinics	8	13.3
On the date of review (6-month review), CDU patients who also must undergo blood testing must queue with the rest of the patients for consultation	8	13.3
Patients´ details on the label are incorrect	3	5
Shortage of CDU parcels received at the clinics and/or pick-up-points	3	5

Overall, Table 4 shows that the level of CDU service delivery was perceived as effective across the four clinics, with the majority of participants (72% +) answering affirmatively with regard to the different variables measuring this construct. The only exception was in relation to incidences of parcels not being delivered, as 72.7% of participants (n = 8) indicated that they had encountered this problem. Similar to patient participants, nurses agreed that there has been a marked decrease in patients´ waiting times at all four clinics since the CDU implementation, with 9 out of 11 participants answering affirmatively (81.8%). Table 5 shows that predominant challenges experienced by nurses across the four clinics like “the ineffective system at CDU for notifying clinics of rejected scripts on time” and “CDU parcels not delivered on time” were points of concern about CDU service delivery. In relation to incidences of shortage and late delivery of CDU parcels at clinics, nurse participants (n = 7) stated that they had to dispense medication to CDU-enrolled patients from the limited stock available at the clinic. Challenges linked to CDU processes (experienced by both nurse and patient participants) may necessitate a closer inspection of CDU processes, in order to address shortfalls within the system that may hamper the quality of service delivery.

**Table 4:** nurses´ responses at the four clinics related to CDU service delivery (N=11)

Items	Frequency (n)	Percentage (%)
	**Yes**	**No**
We receive CDU parcels per prescription	10/11 (90.9%)	1/11 (9.1%)
We have had incidences of parcels not being delivered	3/11 (27.3%)	8/11 (72.7%)
We have noticed a decrease in patients’ waiting time at the clinic	9/11 (81.8%)	2/11 (18.2%)
Access to ART through CDU has improved	9/11 (81.8%)	2/11 (18.2%)
We are satisfied with CDU services	8.5/11(77.2%)	2.5/11 (22.8%)

CDU: central dispensing unit, ART: anti-retroviral treatment

**Table 5:** challenges experienced by nurses at the four clinics with regard to the CDU program (N=11)

Challenges	Frequency (n)	Percentage (%)
Ineffective system for notifying clinics of rejected scripts on-time	11	100
CDU parcels not delivered on time	7	63. 6
“Out-of-stock” medication not effectively communicated to clinics	5	45.5
The number of parcels received at clinics does not always equates to the number of prescriptions sent to CDU (i.e. shortage of parcels received at clinics)	4	36.4
Receipt of the wrong medication	3	27.3
Lack of transport to pick-up-points	3	27.3

## Discussion

This study aimed to collect empirical data on the effectiveness of the Germiston CDU in distributing ART to clinics from the perspectives of CDU-enrolled patients and nurses. Four PHC clinics in Ekurhuleni health district in the Gauteng province of South Africa were used as a case study. CDUs play a critical role in alleviating the burden experienced at public healthcare facilities by improving the accessibility of ART and shortening patient waiting time. The main findings showed that the Germiston CDU is essentially “effective” in rendering services to clinics in Ekurhuleni from the vantage points of both patients and nurses. Both groups viewed the level of CDU service delivery as highly satisfactory. They also agreed that there has been a noteworthy decrease in patient waiting times at all four clinics since the implementation of the CDU. Conjointly, these findings showed that the primary motives for the implementation of the Germiston CDU have, largely, been achieved. Despite these positive findings, certain challenges were identified. Both groups of participants indicated that the late delivery of CDU parcels at pick-up points from the clinic was a persistent problem. According to the feedback from nurse participants, there is a limited number of vehicles available at many of the clinics, which may partly explain why parcels are sometimes not received on time. It is recommended that the transport issues at the clinics be resolved.

A main challenge experienced by patient participants was the need for a more convenient collection timeframe. The collection timeframe of 7 am to 9 am for CDU parcels was deemed “too short” as patients do not always arrive on time to collect their treatment, due to challenges such as transport. In addition, other participants expressed that the collection timeframe clashes with their starting time at work. A possible solution here is to consider incorporating weekends to accommodate patients who are unable to timeously collect their treatment on weekdays. In a comparable study, Muthelo *et al*. [16] highlight that in the National Department of Health's Central Chronic Medicine Dispensing and Distribution or CCMDD program in the Vhembe district of Limpopo province of South Africa, patients can access treatment from clinics and various pick-up-points. These include private pharmacies like Clicks and Dischem that have long operating hours on weekdays and weekends. More convenient operating hours could, therefore, help ease access to chronic medicines at facilities.

Similar to the study conducted by Magadzire *et al*. [17], findings from this study showed that the CDU is an essential and innovative way to improve access to chronic medicines. Findings from this study also concurred with results from a study by Du Toit [6,18], which found that centralized dispensing programs such as a CDU have successfully decongested healthcare facilities and enhanced access to chronic medicines. Furthermore, findings from this study´s CDU model mirrored challenges experienced by nurse participants in the study conducted by Muthelo *et al*. [16] referenced above. Shared challenges of the two centralized dispensing programs (CDU and CCMDD) are the late delivery of patient medicine parcels to facilities or pick-up points, as well as the receipt of incorrect medicines.

**Remedial approaches based on specific challenges emerging from the study:** specific challenges like “ineffective notification system related to rejected prescriptions” and “late and/or shortage of CDU parcels delivered” warrant a closer inspection of the Germiston CDU processes. A future endeavor could be to potentially explore this aspect of the study further. For now, as a recommendation, a more strategic and consistent approach to operations can be achieved in the following ways.

Firstly, establishing an efficient notification system with regard to rejected prescriptions is critical. The main challenge unanimously experienced by nurse participants was the ineffective system currently used for notifying clinics about rejected prescriptions, which have consequently led to delays. Prescriptions not adhering to the legal prescription requirements are deemed invalid; subsequently rejected and returned to clinics for rectification. However, tardiness in notifying clinics of rejected prescriptions impedes timeous rectification.

Secondly, to achieve maximum production output and prompt delivery of services, it is important to capitalize on the given lead time by efficiently and strategically working through the workload, while being cognisant of required deliverables. In addition, non-exhaustive actionable steps like setting daily targets for dispensing and strategically allocating staff to key subunits as well as ensuring even distribution of staff at working tables in the dispensary ought to be taken. Moreover, the synchronization of production between key subunits (i.e. VVP and dispensary) will ensure the fluidity and efficiency of CDU operations [19].

Thirdly, another recommendation that would enhance the program´s performance and simultaneously alleviate backlogs that delay the prompt delivery of services is the full (and not partial) implementation of a digital system at the Germiston CDU. Currently, only a fraction of prescriptions received from the enlisted clinics are processed through a digital dispensing program and most prescriptions are dispensed manually. A case study conducted by Magadzire *et al*. [5] postulates that technological advancements attributed to the remarkable expansion of the CDU scope in the Western Cape Province. As a result, there was an upsurge in production output. Therefore, a digital system would ensure a speedy dispensing process with more accuracy and reliability, thus minimizing manual errors such as incorrect patient details on labels [20,21]. This would also facilitate the inclusion of all 93 clinics (as opposed to the current select few) into RxSolution software used at Germiston CDU.

Fourthly, the establishment of a robust workforce at the Germiston CDU is crucial. Although the lack of a robust workforce was not highlighted as a main challenge by nurse participants as contributing to backlogs, anecdotal evidence at the time of data collection suggested that the late deliveries of medicines experienced by nurse participants (Table 5) could have partly been attributed to the lack of a strong workforce. It was also mentioned that learner pharmacist assistants make a meaningful contribution to the productivity of the program. Conversely, the pressure and burden of workload are often felt when contracts terminate. Since permanent employees are generally the cornerstone of an organization's workforce, reliance is not to be heavily placed on contract staff.

Lastly, incorporating an operational pharmacist into the organogram may be a constructive addition to the current operational structure. An operational pharmacist can aid in the smooth running of operations. The role of the operational pharmacist must be well-defined; strategically aimed at ensuring operational efficiency and consistency.

**Limitations:** given that the study was small in scale with a sample size of 60 patient participants, the findings could not be generalized and assumed to represent all 93 clinics receiving services from the Germiston CDU. However, it must be noted that the intention of the study was not to generalize, but rather to conduct a preliminary district analysis for quality improvements. A further limitation relates to the perspectives of former CDU patients that could not be obtained due to the transfer of these patients from the CDU to CCMDD during the time of the study. Data from these patients could have provided more insight into the research.

## Conclusion

In general, the Germiston CDU was deemed effective in rendering services to clinics in the health district of Ekurhuleni. However, the viability of the CDU relies on the development of a strategic and consistent approach to operations, to ensure optimisation of productivity, as well as maintain efficient service delivery. Building on the successes as well as the challenges identified in this study can help guide future plans and policies. The Germiston CDU is an essential public-sector dispensing and distribution program that provides value-added services to patients on long-term therapy. Therefore, for a decanting program of this magnitude to successfully meet its objectives, it is imperative to considerably invest in its input and processes.

### What is known about this topic

- *A CDU was first introduced in the Western Cape Province in 2005 as an innovative way to enhance access to chronic medicines for patients on long-term therapy;*
- *With increased demand to decongest healthcare facilities in Ekurhuleni Health District, the Germiston CDU was implemented in 2015;*
- *The centralized dispensing programs have significantly contributed to the decongestion of healthcare facilities and enhanced patients´ accessibility to chronic medicines*.

### What this study adds

- *In light of the paucity of empirical studies on the effectiveness of a CDU from the vantage point of patients, this study adds new, much-needed information in this regard;*
- *This study provides baseline information for the development of a monitoring and evaluation tool on service delivery for centralized dispensing programs*.
